# Research Trends, Biases, and Gaps in Phytochemicals as Insecticides: Literature Survey and Meta-Analysis

**DOI:** 10.3390/plants12020318

**Published:** 2023-01-10

**Authors:** Lara J. Collares, Leonardo M. Turchen, Raul Narciso C. Guedes

**Affiliations:** 1 Departamento de Entomologia, Universidade Federal de Viçosa, Viçosa 3657-900, MG, Brazil; 2 Neo Ventures, Rua Alameda Vicente Pinzon, 54, 9° Andar, Vila Olímpia, São Paulo 04547-130, SP, Brazil

**Keywords:** arthropods, bioinsecticides, botanical insecticides, insect pest management, pest control, natural products

## Abstract

A 76-year literature survey and meta-analyses were carried out to recognize the trends, biases, and knowledge gaps of studies focusing on major groups of compounds of botanical origin, or phytochemicals, as insecticides. The survey found that the main phytochemicals prospected as insecticides belong to the following major chemical groups: terpenoids, terpenes, and carbonyl, all of which were tested, mainly against beetles (Coleoptera), caterpillars (i.e., larvae of Lepidoptera), and mosquitoes and other flies (i.e., Diptera). These studies are burgeoning at an exponential rate, with an evident focus on mortality endpoint estimates, but they are also neglecting sublethal assessments. China and India in Asia, as well as Brazil in the Americas, were responsible for most studies. The majority of the papers used stored grain insects as experimental models, which limits the applicability and representativeness of the findings. As a result, the main modes of exposure tested were fumigation and contact, which leads to the prevalence of estimates of lethal concentration in these studies. Therefore, a broader range of insect species deserves testing, with suitable modes of exposure identifying and characterizing the main molecules responsible for the insecticidal activity, which is seldom performed. Attention to these needs will circumvent current biases and allow the recognition of the main patterns of association between the origin and structure of phytochemicals and their insecticidal effects.

## 1. Introduction

The Earth is blue, but the world is green! The former is the natural consequence of the ¾ths coverage of the planet surface by the oceans, while the latter statement is the result of the prevailing coverage of the Earth’s landmass by plants with their chlorophyll-green reflected light. Yet, where there are plants, insects exist and prevail as the most ubiquitous animal occurrence on Earth. Curiously, plants are the original source of food for microbial and animal life, insects included, but are not consumed to the point of their demise. Thus, the question—“*Why is the world green?*” was put forward as early as the 1960 [1]. This question was tested, contested, discussed, and found to be productive, leading to the onset of the plant defense theory [1,2,3,4]. The world remains green, and insects, particularly herbivore insects, still abound. Therefore, plant defense against herbivores and plant–insect interaction remain relevant fields for answering broad eco-evolutionary questions [5,6,7,8]. The Anthropocene and the onset of agriculture some 13,000 years ago provide additional ingredients to this scenario [9,10], as crop domestication compromised genetic diversity for resistance to insect herbivores and changed plant resource allocation from defense to yield. The result was the increasing vulnerability of crop plants to phytophagous insects and the shifting of their status to that of pest species in need of (artificial) control [11].

The creation of the Garden of Eden may not have required the use of pesticides [12], but agriculture intensification and crop vulnerability to pest species drove the need for insect control, in which natural compounds were an early feature, subsequently replaced by synthetic insecticides [13,14]. The latter remains a prevalent agent of pest control [14,15,16,17], but are fraught by enduring controversies regarding their health and environmental safety, further amplified by shifting societal concerns and regulatory restrictions [18,19,20,21,22]. Thus, attention is steadily changing towards natural compounds to be used as insect pest control agents, not only due to their early prominence before the onset of synthetic pesticides in the late 1940’s [23,24,25,26], but also as the backbone and source of inspiration for the design of novel pesticides, particularly new insecticides [27,28,29,30,31].

The ongoing public pressure for safer pesticides led to new paradigms and policies that promote the search for and use of biorational insecticides, or bioinsecticides, based on their perceived advantages [32,33,34]. Such advantages include low environmental and mammalian risk, higher specificity and safety to non-target organisms, lower risk of resistance development, and lower persistence, despite the incipient experimental evidence of such perception [13,35,36]. Botanicals are at the forefront of this scenario and receive the bulk of the attention [24,35,37,38,39]. However, this increased attention is not resulting in increased usage [40], and the accumulated data are not generating as much information [41], which is channeled to a limited group of plant species, with prevalence of Meliaceae and a dozen additional families (e.g., Lamiaceae, Asteraceae, Myrtaceae, Rutaceae, etc.), as well as a few target arthropod species, mainly pest species. There is reliance on mortality assessments, neglecting other important insecticidal effects, such as decreased reproduction and repellence [12].

Furthermore, another shortcoming regarding the studies on botanical insecticides is the fact that the focus is on the plants themselves and their extracts, while limited information is provided on the (phyto)chemicals involved with insecticidal potential. The cultivation context, plant genotype, extract preparation, and the pattern of usage affect the level of phytochemicals present. Thus, the recognition and quantification of the phytochemicals involved is paramount and has attracted great interest lately. However, if one wishes to understand where good ideas come from, context is necessary to provide meaning and to recognize and solve shortcomings. Previous (qualitative) reviews recognize the importance of phytochemicals in plant extracts as insecticidal compounds, but they lack a quantitative approach and testing to identify existing trends, bias, and knowledge gaps, which has motivated the present effort.

Here, a systematic literature survey on the subject was carried out to draw a timeline of the studies as the first step to understand the subject, its state-of-the-art status, and limitations. The survey was performed using the Web of Science database, spanning from its inception year, 1945, to March 2021. The data-gathering allowed meta-analyses to integrate the data and perform the testing, with the objectives of recognizing (i) the overall publication trends, (ii) the focus of attention and main contributors, and (iii) the possible biases and existing knowledge gaps that deserve future attention.

The prevalence of plant species of Meliaceae in studies on botanical insecticides is suggestive that the prevailing phytochemicals in these species are the main focus of the growing number of studies on phytochemicals as insecticides [12]. Essential oils are also emphasized in qualitative reviews, which suggests that their components are also the focus of attention as phytochemicals [12,35,38,39,42]. The same insect orders targeted in studies with botanical extracts are expected to prevail in studies with isolated phytochemicals [12,35], and such a relationship is likely directing the type of testing of biological activity carried out in the studies. This information will provide a better understanding of the subject and guide further research efforts, which justifies the present study.

## 2. Results

### 2.1. Summary of the Literature Surveyed

**Figure 1:**
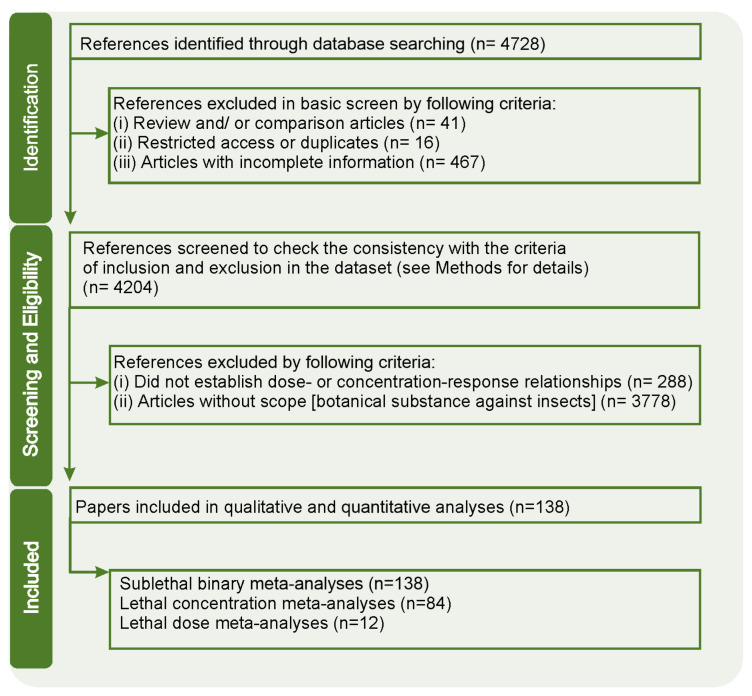
Flowchart diagram describing the steps of filtering data from scientific papers obtained from the systematic literature survey review that used the Web of Science database (1945–April 2021).

The literature survey on the Web of Science database resulted in 4728 articles, but 524 of them were excluded in the preliminary screening, 3778 were excluded because they did not meet the study scope, and 288 were excluded because they did not establish a dose– or concentration–response relationship. Thus, 138 papers were deemed suitable for the purposes of the desired qualitative and quantitative analyses (Figure 1; Supplementary Materials).

### 2.2. Qualitative Trends: Study of Geographical Origin, Plants, Insects, and Phytochemicals

**Figure 2:**
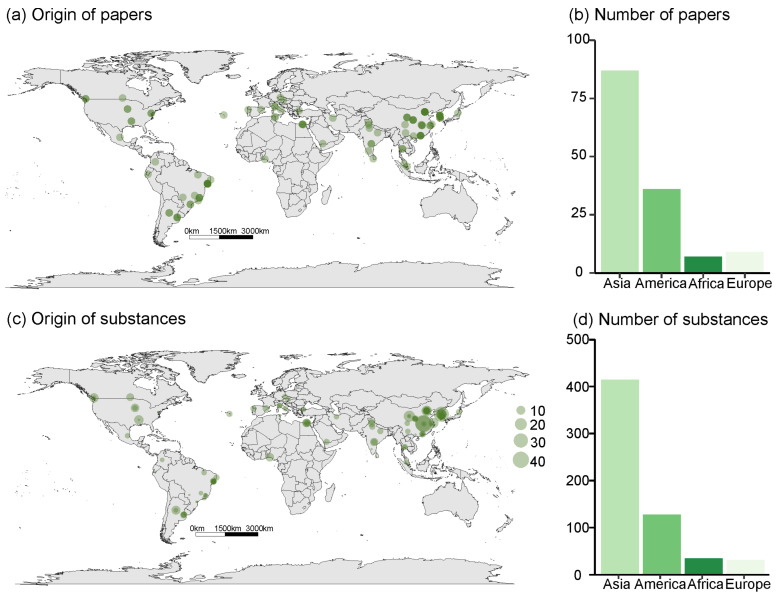
(**a**) Maps and bar plots identifying the geographic distribution of the first author of published articles. (**b**) Number of works produced by authors from each continent. (**c**) Bubble chart map indicating the number of substances studied by region. (**d**) Number of times that substances of botanical origin were studied by authors from each continent.

The authors of studies evaluating the effects of insecticidal molecules of botanical origin were mainly from Asia, especially China. The results show that Asia is the continent with the largest number of leading researchers and substances studied. The Americas are also well-represented by leading researchers, particularly in Brazil. In contrast, leading researchers from Africa and Europe published little on the subject, with only seven and eight articles from these continents, respectively. None of the studies analyzed originated in Oceania (Figure 2).

Although Europe publishes more than Africa, African publications have investigated a greater number of substances per publication. The average number of substances studied per article in the world is 4.19, while in Africa, this average is 5; in Asia, it is 4.8; in Europe, 3.4; and in America, only 2.5 substances are studied per article, on average (Figure 2).

**Figure 3:**
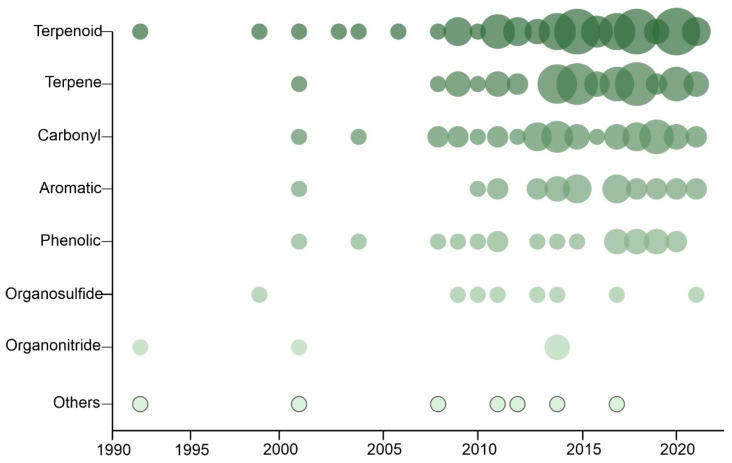
Scatterplot of number of articles with the chemical group published per year. The circle size is proportional to the number of articles published per year with each class of phytochemicals, and the circle color darkens with the largest overall number of articles published on each phytochemical.

The first article in the period of analysis was published in 1993 without any further article published for the next five years; 84% of all articles analyzed were published after 2010 (Figure 3). Terpenoids were the class of insecticidal phytochemicals most studied so far (34% of the studies), followed by terpenes (23.2%) and carbonyl (16.4%). The least-studied classes were organonitrides (2%) and others (2.8%).

**Figure 4:**
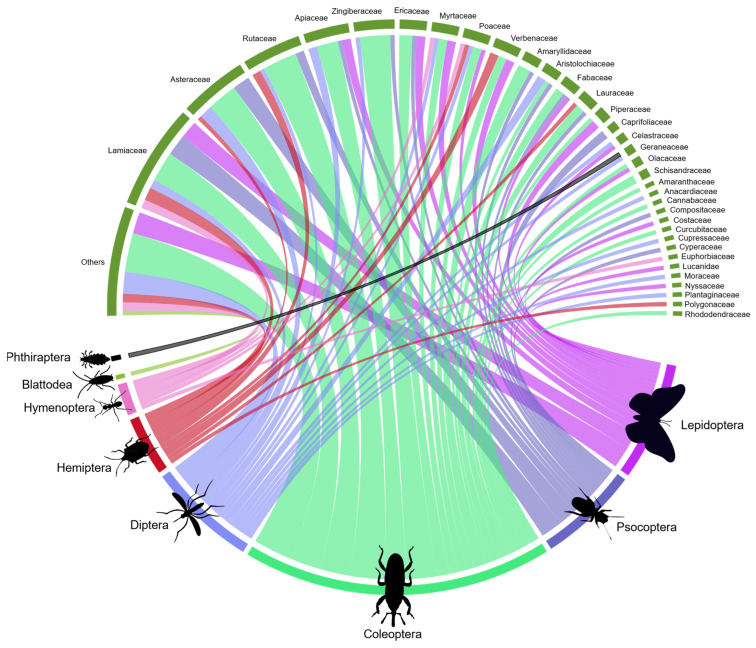
Interaction between the diversity of plant families and insect orders studied from the bibliographic survey of articles on insecticidal phytochemicals. The thickness of the bar and line under each plant family and insect order connecting them corresponds to the relative number of articles dealing with the said insect groups and molecules derived from plant groups; the thicker the bar and line, the larger the number of studies. (Number of papers = 138. However, there are repeated measures, since a paper may contain more than one insect order and/or botanical family; therefore, n = 162).

The 138 studies tested insecticidal phytochemicals on eight insect orders, focusing mainly on beetles (i.e., insects of the order Coleoptera; 42%), followed by butterflies and moths (i.e., Lepidoptera; 17%) and mosquitoes and other flies (Diptera; 16%). Compounds from 34 botanical families were studied. Lamiaceae, Asteraceae, and Rubiaceae were the most well-represented families, respectively (Figure 4). Overall, the studies were diverse and tested compounds originating from different families in the same insect order. 

**Figure 5:**
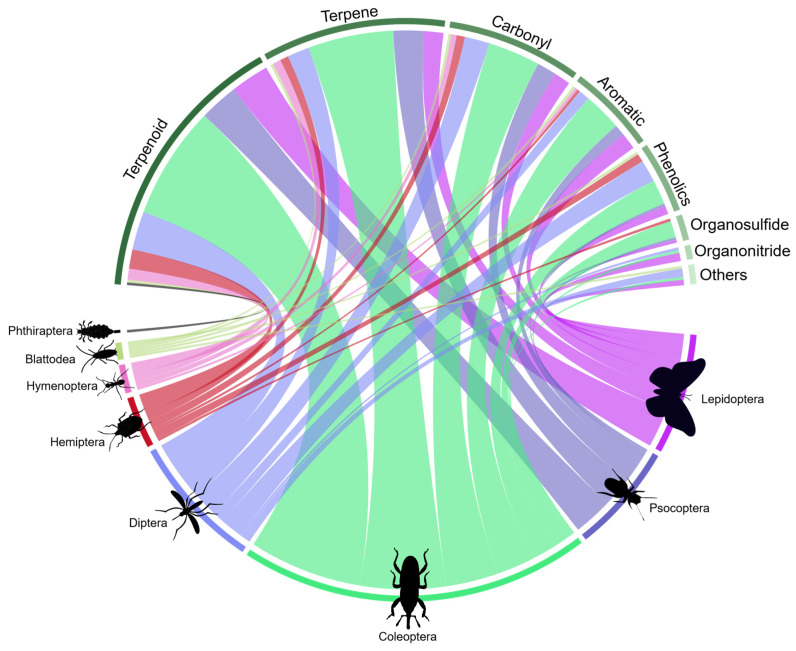
Interaction between the diversity of insecticidal groups of phytochemicals and the orders of insects tested based on the literature survey. The thickness of the bar and line under each chemical group and insect order connecting them corresponds to the relative number of articles dealing with the said insect groups and chemical groups; the thicker the bar and line, the larger the number of studies. (Number of papers = 138. However, there are repeated measures, since a paper may contain more than one insect order and/or chemical groups; therefore, n = 284).

Eighty-seven percent (87%) of the studies assessed phytochemicals belonging to the classes terpenoid, terpene, carbonyl, or phenolic. Out of these, 73% were terpenoids or terpenes, representing the focus of attention of such studies. The main insect orders studied were Coleoptera (43%), Diptera (17%), Lepidoptera (15%), and Psocoptera (13%), which correspond to 88% of the studies and encompass all phytochemical groups (Figure 5).

**Figure 6:**
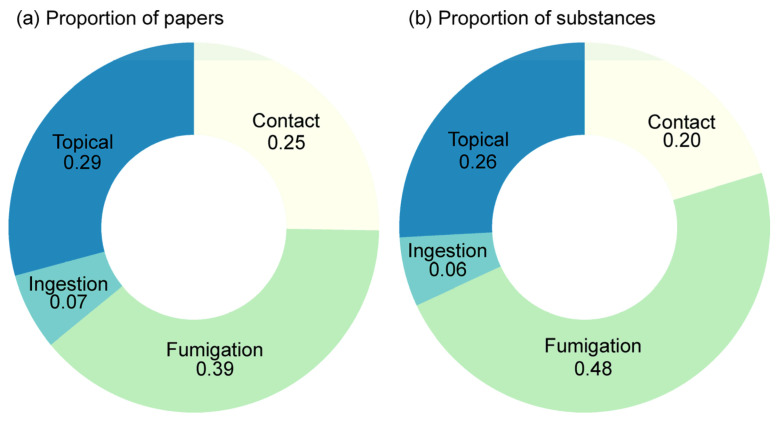
Types of exposure route present in the experiments of the analyzed articles. (Number of papers = 138; however, there are repeated measures, since a paper may contain more than one exposure route or substances. Therefore, n = 178 and n = 1121 for (**a**) and (**b**), respectively).

Fumigation was the most frequent method of exposure observed in the studies screened, followed by topical application, contact, and, finally, ingestion. Fumigation was used for testing almost half of the analyzed substances (48%) and was present in 39% of the articles surveyed (Figure 6).

### 2.3. Quantitative Trends: Meta-Analyses

**Figure 7:**
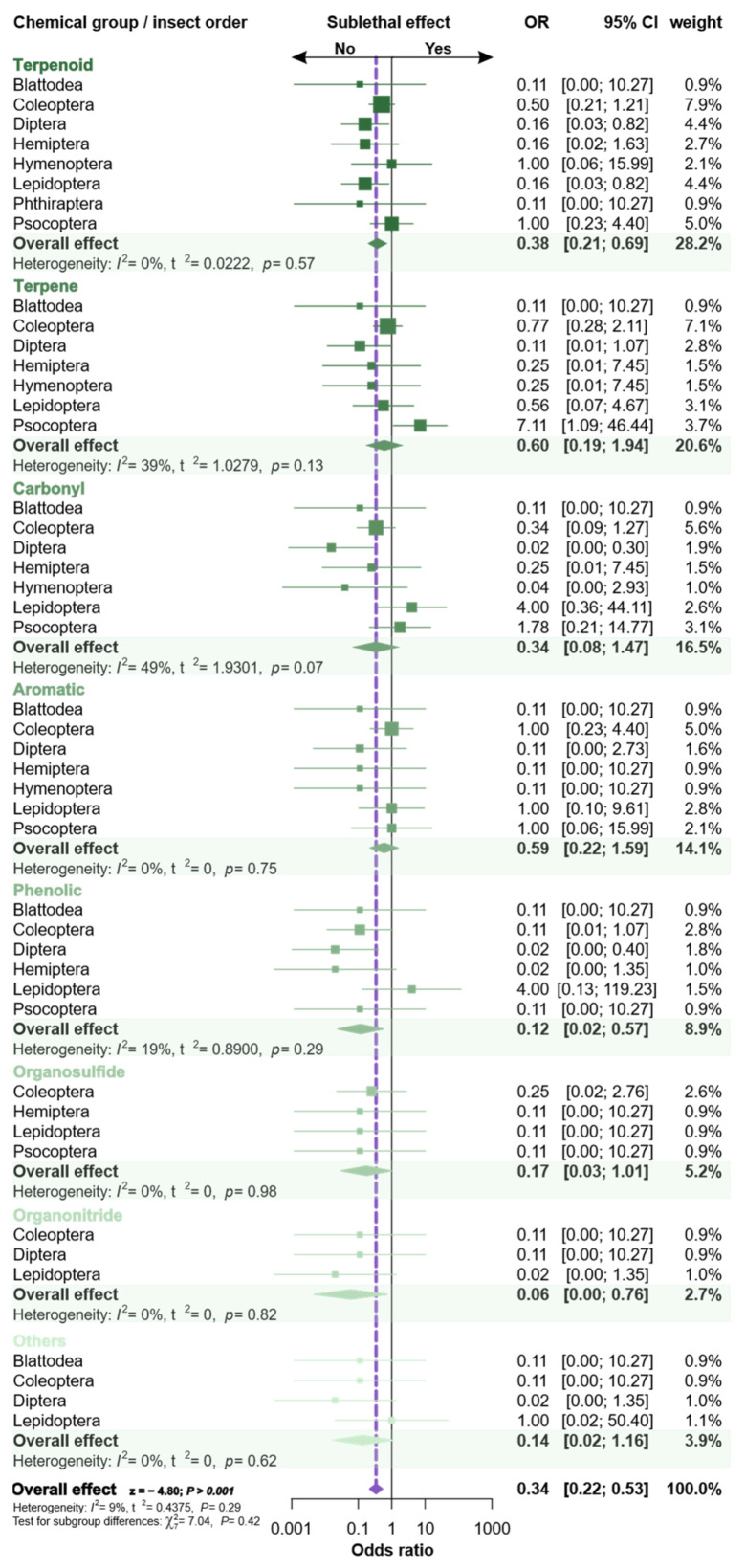
Forest plot summarizing the binary meta-analysis evaluating the existence or not of sublethal assessment in articles on the insecticidal activity of phytochemicals. The meta-analysis is divided into phytochemical groups and insect orders (subgroups). The proportion is denoted by colored boxes and the 95% CIs are the horizontal lines. The overall trends are represented by a colored diamond, where the diamond width corresponds to 95% CI bounds. The vertical dashed line shows the overall estimated effect resulting from all studies. The *p*-values for the heterogeneity test are indicated (number of papers = 138).

The low incidence of sublethal response assessments in the surveyed studies with insecticidal phytochemicals was subjected to meta-analyses to detect whether the perceived bias was restricted to some insect orders and/or some chemical groups of substances, or if it was a general trend (Figure 7). The global effect estimated by the meta-analysis confirmed that the sublethal assessment of phytochemicals was rarely considered or tested (i.e., RR = 0.34; z = −4.8; *p* > 0.001). When the phytochemical subgroups are individually considered (terpenoids, phenolics, and organonitrides), it is statistically evident that the sublethal effects are not a target of attention, while the combined estimates of the other chemical subgroups were not statistically significant either. Thus, the insecticidal assessment of phytochemicals is significantly biased towards mortality assessment, without considering alternative toxic responses.

The overall effect of the dataset exhibited low heterogeneity between chemical groups (I^2^ = 9%). However, a median heterogeneity between 19% and 49% was observed in the subgroup of terpenes, carbonyl, and phenolics, since the studies evaluating this effect were carried out mainly among butterflies/moths and booklice (Lepidoptera and Psocoptera, respectively).

**Figure 8:**
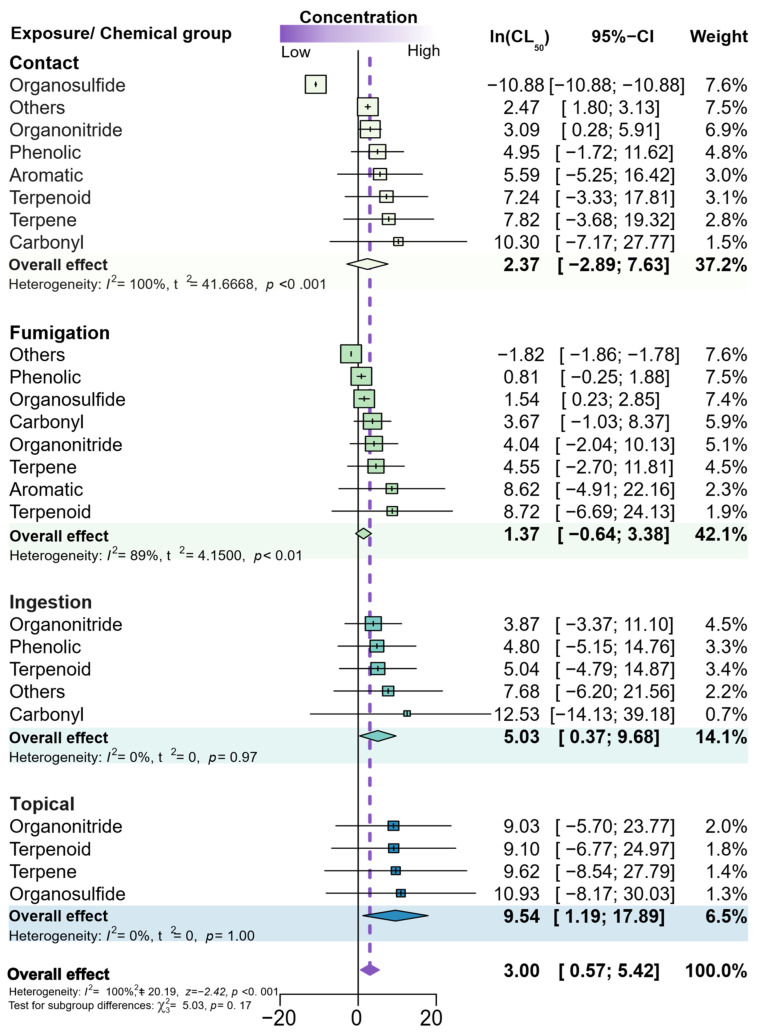
Forest plot summarizing the quantitative meta-analysis comparing the LC_50_s, as toxicity endpoints, in papers assessing the insecticidal activity of phytochemicals. The meta-analysis is divided into modes of exposure and phytochemical group. Concentrations (ppm) were transformed by natural logarithm. The average LC_50_s are denoted by boxes (horizontal lines), and the box size refers to the number of populations reported for the phytochemical group; the 95% CIs are represented by horizontal lines. The combined LC_50_ estimate for overall trends is represented by a diamond, where the diamond width corresponds to 95% CI bounds. The vertical dashed line shows the overall estimated effect resulting from all species. The *p*-values for the heterogeneity test are indicated (number of papers = 84).

Additional meta-analyses were performed to recognize whether the phytochemical insecticidal potency, represented by its LC_50_, varied with its mode of exposure, if by contact, fumigation, ingestion, or topical exposure. The general trends estimated via a random model meta-analysis (see purple diamond) demonstrate that the lethal concentrations were higher than 1 ppm—about 20 ppm, in fact (Ln_[general effect]_ = 3.00), which indicates a relatively low potency (Figure 8).

Phytochemical toxicity, measured as the LC_50_ as the toxicological endpoint, did not vary significantly among modes of exposure (Figure 8). When the individual modes of exposure were considered, rather than the overall trend, topical exposure and ingestion followed the overall effect, without exhibiting significant toxicity differences among the phytochemical groups. Nonetheless, such toxicity differences were significant among phytochemicals for fumigation and contact exposure, and the effect of the different chemical groups was heterogeneous (100% and 89%, respectively).

**Figure 9:**
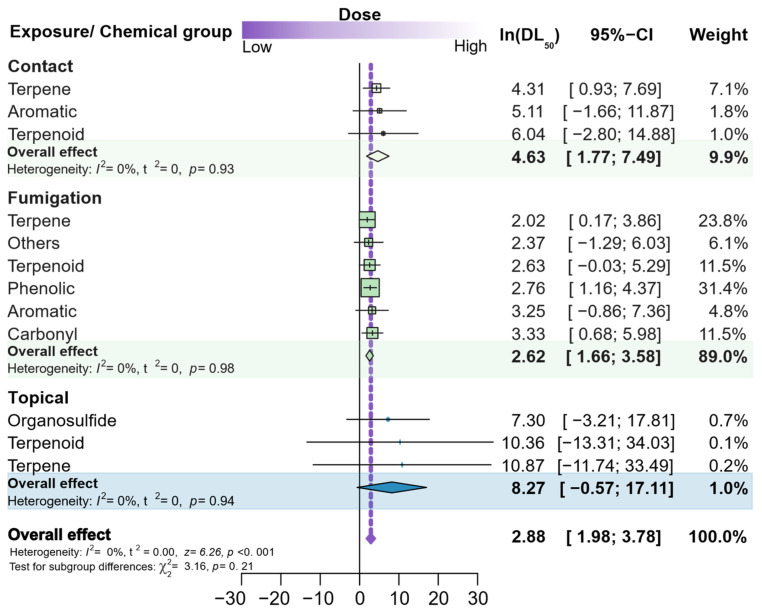
Forest plot summarizing the quantitative meta-analysis comparing the LD_50_s, as toxicity endpoints, in papers assessing the insecticidal activity of phytochemicals. The meta-analysis is divided into modes of exposure and phytochemical group. Doses (ppm) were transformed by natural logarithm. The average LD_50_s are denoted by boxes (horizontal lines), and the box size refers to the number of populations reported for the phytochemical group; the 95% CIs are represented by horizontal lines. The combined LD_50_ estimates for overall trends is represented by a diamond, where the diamond width corresponds to 95% CI bounds. The vertical dashed line shows the overall estimated effect resulting from all species. The *p*-values for the heterogeneity test are indicated (number of papers = 12).

The last meta-analysis was carried out with the same purpose as the previous one, but this time, it aimed to recognize whether the toxicity determined through LD_50_ estimates, rather than through LC_50_s, of insecticidal phytochemicals (represented by their chemical groups) differed according to the mode of exposure. As for LD_50_, the general trends estimated using random model meta-analyses (see purple diamond) revealed that the median lethal doses of phytochemicals were higher than 1 ppm—about 18 ppm (Ln_[general effect]_ = 2.88), regardless of the mode of exposure, which had a negligible effect (Figure 9). The overall toxicity among the phytochemicals did not differ among the modes of exposure, nor did they differ among phytochemicals within each mode of exposure, despite the significant variation among the modes of exposure. Therefore, when each mode of exposure was individually considered, the response to fumigation was prevalent and accounted for 89% of the determinations, thus limiting the representativeness and resolution of the estimates.

## 3. Discussion

The present investigation was motivated by the increasing interest in bioinsecticides, particularly botanical insecticides, based on the shifting of societal and regulatory concerns, coupled with the lack of quantitative analyses of the publication trends exploring such compounds. A previous study focused on botanical insecticides in the form of raw extracts [12]. However, plant compounds themselves, or phytochemicals, have not been targeted yet, which led to the present effort. Thus, a systematic literature survey and meta-analyses were carried out on the subject, aiming to recognize (i) the overall publication trends, (ii) the focus of attention and main contributors, and (iii) the possible biases and existing knowledge gaps deserving further attention. A convergent attention to plant species of Meliaceae and their phytochemicals was initially expected, due to the prevailing focus on this plant family in studies with raw botanical extracts [12]. Phytochemical components of plant essential oils were also generally expected to be the center of interest, due to the attention given to such oils [12,38,39,42]. The insects targeted in such studies on insecticidal activity were also expected to be similar to those with plant extracts [12,35], which would affect the prevalent types of testing used to assess the biological activity of phytochemicals. Interestingly, the observed results did not meet such expectations.

An initial noteworthy finding of the literature survey is the scarcity of studies on botanical insecticidal compounds that recognized and targeted phytochemicals as biologically active compounds against insects; only 138 studies, out of the initial pool of 4728 (i.e., about 3%). This concern has already been expressed [41] and remains valid. Therefore, the potential of phytochemicals as insecticides and particularly as the backbone and source of inspiration for the design of novel insecticides remains wholly neglected, which deserves attention. A likely explanation for the detected scenario is the timeline of the evolution of studies targeting phytochemicals as insecticidal compounds, which is a recent trend that started only in 1993. This perception is further reinforced by the fact that 84% of the studies surveyed consistent with our criteria of assessment were all published after 2010. Thus, they were published much later than the studies with botanical insecticides, which date back to the mid-1940s [12,35,37,41]. The popularization of chromatography and chromatographic techniques observed since the 1990s probably contributed to the recent expansion of the identification and characterization of insecticidal phytochemicals [43]. 

Two trends are relatively easy to perceive regarding botanical insecticides. The first is the increasing societal demand for safer agrochemicals, particularly pesticides, which reflects priority shifts in pesticide regulation, especially for agriculture. The European Union is prominent in this scenario and has set high demands and expectations for biopesticides [33,34], but this emphasis resonates in the regulatory agencies of different nations [32,36,38]. The second trend refers to the increasing complexity of the studies on botanical insecticides, which usually require suitable equipment and expertise in time-consuming and multidisciplinary efforts [37,44,45]. Such studies are usually associated with wealthier members of the Organization for Economic Co-operation and Development (OECD), such as the US, EU members, Japan, Australia, and Canada. Nonetheless, three non-OECD members retain historical dominance in studies addressing botanical insecticides—China, India, and Brazil [41]. The same trend is apparent in studies with phytochemical insecticides, as recorded here. The diversity of plant species of a country and their traditional use are potential explanations for that. However, at least regarding China, the national contribution to research and patents on bioactive phytochemicals also significantly benefits from governmental, economic, and bureaucratic incentives [46]. This will potentially favor these three leading countries in insecticidal phytochemical research to establish promising markets for the product of their efforts.

Meliaceae is the main plant family targeted in studies on botanical insecticides, followed by Lamiaceae, Asteraceae, and Myrtaceae, approached in studies aimed mainly at pest species of Coleoptera, Diptera, and Lepidoptera (i.e., beetles, mosquitoes and other flies, and moths and butterflies (or their larvae, more precisely)) [12,35]. Even for non-targeted organisms, Meliaceae is also the most-studied plant family as a source of botanical insecticides [12]. Although beetles, mosquitoes and other flies, and caterpillars of moths and butterflies are also the main insect groups investigated by studies on insecticidal phytochemicals, Meliaceae plants are not a significant target source of chemicals, unlike Lamiaceae, Asteraceae, and Rutaceae, keeping these three main plant families serving this purpose. The reasons for that are unclear, especially considering that the limonoid azadirachtin, a well-known triterpenoid obtained from the neem tree (*Azadirachta indica* A. Juss), a Meliaceae, is the most widely explored botanical compound in the world [35,41]. The insect species, rather than orders or families, may hold insights to explain that though.

Terpenoids, terpenes, and carbonyl are the phytochemical groups most explored for insecticidal activity, and the two former ones account for ¾ of the studies. The prominence of the compound azadirachtin is likely one of the reasons for that, but there is more to it. Again, beetles, mosquitoes and other flies, and caterpillars of moths and butterflies were the main insect groups tested in that regard, curiously followed by booklice, which do not feature among the main insect orders targeted in studies of (raw) botanical insecticides [12]. The dominant use of fumigation as mode of exposure accounts for nearly half of the studies of insecticidal phytochemicals and provides important clues for a few existing biases among the surveyed studies—the targeting of insect pest species in such studies, rather than natural enemies or pollinators, and the significant focus on stored product insect pest species.

The reliance on insect pest species in the search for insecticidal phytochemicals is expected, since the control of these organisms is the objective of the studies surveyed. However, the assessment of non-target effects is an important component for the development of suitable insecticides for pest management within the current context of enhanced concerns regarding the environmental safety of these molecules. This deficiency was also recognized for biopesticides at large [35] and botanical insecticides in particular [12]. Regardless, the overemphasis on stored product insect pests as experimental models for assessing insecticidal phytochemicals was not expected. That said, such use makes sense, since this group of pest species is important, well-known biologically and behaviorally, usually inexpensive to rear, and includes individuals with short life cycles [47,48,49,50]. The phasing out of methyl bromide as a fumigant due to its ozone-depleting characteristics [51] and the overreliance on phosphine as the dominant worldwide fumigant with ever-increasing problems of phosphine resistance [52], in addition to public health concerns with its use [53,54], provided further incentive for the search of botanical fumigants. Nonetheless, the standing prevalence of stored product insects in studies of insecticidal phytochemicals and fumigation activity compromises the representativeness and reach of the studies on this subject.

The studies on insecticidal phytochemicals surveyed also hold additional biases worthy of consideration. Foremost among them is the reliance on lethal or mortality assessment and related toxicological endpoints—the median lethal dose/concentration, which permeates all phytochemical groups and insect orders assessed. This bias arguably prevents the recognition of potentially valuable insecticidal phytochemicals that minimize pest losses by means other than causing direct pest mortality (e.g., by sterilization, delayed development, repellency, and phagodeterrence of insect pests). Again, this is mirroring a common shortcoming among studies with biopesticides and even conventional insecticides [12,14,55,56,57,58], which also compromises the understanding of the non-targeted effects of the insecticidal compounds [55,58,59,60].

The last issue of concern regarding the existing studies on insecticidal phytochemicals is their relatively lower potency and the intriguing factor that, among the most-studied phytochemical groups—terpenoids, terpenes, and carbonyl—toxicity tends to be somewhat low when compared with conventional insecticides, and even more so when fumigants are considered. Thus, the broadening of the scope of plant sources, phytochemical groups and compounds, pest species targeted and toxicological endpoints considered seems paramount for further and better-oriented efforts regarding insecticidal phytochemicals. The eventual testing of binary or multi-component phytochemical mixtures should not be neglected either, as their co-occurrence in plants is the norm, not the exception, and their interactive effects may result in the potentiation of their insecticidal activity. This is an issue frequently neglected in studies on insecticidal phytochemicals. Thus, there is no shortage of opportunities ahead, all worthy of attention—fiat lux!

## 4. Materials and Methods

### 4.1. Data Collection

The systematic literature survey and subsequent meta-analyses followed the PRISMA guidelines (i.e., Preferred Reporting Items for Systematic Reviews and Meta-Analyses) [61]. The approach followed the steps of identification, screening/elimination, eligibility, and inclusion. The database used was Web of Science from 1945 to April 2021, using the following keywords as search parameters: “bioinsecticide” or “extract” or “phytoinsecticides” or “essential oil” or “natural molecules” or “natural compounds” or “botanical insecticide” or “pesticide”, always in combination with “insecticidal activity” or “insecticide” and “insect”. Only scientific articles published in English and in peer-reviewed journals were considered.

### 4.2. Screening

The initial compiled dataset was used for a basic screening, which adopted the following exclusion criteria: review and/or comparison articles; articles with restricted access or duplicates; articles with incomplete information (e.g., without DOI, plant family and insect order identification). Subsequently, the obtained articles were further scrutinized to exclude (i) studies that did not establish or present dose–response and/or concentration–response relationships; (ii) articles without sufficient statistical data, and (iii) articles not relevant to the research objectives targeted here.

### 4.3. Data Extraction

The following information was recorded for each publication: DOI, title, publication date, geographical coordinates of the article’s 1st author’s address, plant species from which the substance was obtained, plant family, phytochemical studies, insect species targeted, insect order, occurrence of sublethal effect, route of exposure, toxicological endpoint and respective 95% confidence interval, and number of subjects in the control treatment.

The recorded phytochemicals were classified following the group division earlier proposed [62], with minor adjustments, as indicated in Table 1. The main groups of phytochemicals were: phenolics, terpenes, terpenoids, organonitrides, and organosulfides; non-phenolic aromatic compounds and carbonyl were also added as individual groups, due to their significant incidence. The remaining phytochemicals were grouped as “others”.

All the articles selected in the screening processes detailed above were used in the qualitative analyses. One hundred thirty-eight articles were used for quantitative binary meta-analysis, but only the articles reporting toxicological endpoints with units converted to ppm, 94 papers in total, were used in the quantitative meta-analyses to allow comparisons and testing.

### 4.4. Statistical Analyses

Binary meta-analysis was used to test the likelihood of sublethal assessment in contrast with mortality assessments, where the odds ratio and 95% confidence intervals were used to determine the overall effect measured. The odds ratio is the probability of an outcome between two alternatives. These estimates were subject to meta-analysis using a random effect model, considering the phytochemical class as group and insect order as subgroup. The quantification and heterogeneity test and mode of exposure were used as group and the phytochemical class as subgroup. The quantification and heterogeneity tests (i.e., Q, H, and I^2^) were carried out, and the inverse variance and DerSimonian–Laird methods were used to estimate the between-study variance (τ^2^). Studies with n ≤ 1 event in both groups were excluded from the meta-analyses. 

Two additional quantitative meta-analyses were used to quantify the median lethal doses and concentrations among different exposure methods and chemical groups. The median lethal dose (LD_50_) or lethal concentration (LC_50_) and 95% confidence intervals were used to determine the overall median values for the exposure methods and chemical groups assessed. In this case, the LC_50_ or LD_50_ data were converted to units of ppm and transformed by natural logarithm to allow for comparison. 

All analyses were carried out using software version R. 3.5.1 (R Development Core, Vienna, Austria) with the packages “*meta*”, “*metafor*”, and “*stats*”. The graphical illustrations (qualitative and quantitative) were produced with a Wacom creative tablet (Intuos S, Tokyo, Japan), using Corel Painter (Essential 7, Ottawa, ON, Canada).

## 5. Conclusions

The 76-year literature survey and meta-analyses of phytochemical insecticides indicated three main groups of phytochemicals prospected—terpenoids, terpenes, and carbonyl, which were all tested mainly against beetles, caterpillars of moths and butterflies, and mosquitoes and other flies. These studies are burgeoning at an exponential rate, with evident focus on mortality endpoint estimates while neglecting sublethal assessments. China, India, and Brazil were responsible for most of the studies. Most papers used stored grain insects as experimental models, which limits the applicability and representativeness of the findings with focus on fumigation activity. Therefore, a broader range of plant sources, phytochemical groups, insect species, and toxicological endpoints is necessary to circumvent the existing biases and allow further progress in the development of phytochemical insecticides.

## Figures and Tables

**Table 1:** Classification of the phytochemicals recognized in the literature survey and used in the meta-analyses.

Structure	Class	Subclasses [Representative Constituents]
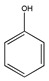	Phenolics	***Aromatic acids*** [e.g., simple phenols, phenolic acids, phenolic aldehydes, etc.] ***Polyphenols*** [e.g., flavonoids, isoflavonoids, curcuminoids, tannins, etc.]
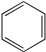	Aromatics	***Non-phenolic aromatics*** [e.g., benzoquinones, acetophenones, phenylacetic acids, coumarins, etc.]
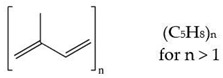	Terpenes	From ***monoterpenes*** [e.g., limonene], to ***diterpene*** [e.g., retinol, phytol], to ***sesquiterpenes*** [e.g., farnesol, humulene], up to ***polyterpenes*** [e.g., natural rubber]
Terpene ((C_5_H_8_)_n_ for n > 1) derivatives with additional functional groups	Terpenoids	From ***hemiterpenoids*** [e.g., isoprenol, prenol], to ***monoterpenoids*** [e.g., camphor, carvone, menthol], up to ***polyterpenoids***
Diverse nitrogen-containing compounds	Organonitrides	***Alkaloids*** [e.g., nicotine, caffeine, morphine, ***Cyanogenic glucosides*** ***Nonprotein amino acids*** [e.g., canavanine]
Sulfur-containing compounds (non-amino acid)	Organosulfides	[e.g., allicin, alliin, piperine, phytoalexins]
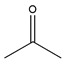	Carbonyl	***Aldehydes*** [e.g., citral], ***ketones*** and ***other carbonyl compounds*** not represented in the previous classes
	Others	[phytic acid, oxalic acid, tartaric acid, malic acid, quinic acid, etc.]

## Data Availability

Available as Supplementary (Excel) File (see Supplementary Materials) upon request to the authors ) upon request to the authors.

## Supplementary Materials

The following supporting information can be downloaded at: https://www.mdpi.com/article/10.3390/plants12020318/s1, Supplementary File S1: Surveyed papers and main data.
